# Effects of neonatal inhalation exposure to ultrafine carbon particles on pathology and behavioral outcomes in C57BL/6J mice

**DOI:** 10.1186/s12989-019-0293-5

**Published:** 2019-02-18

**Authors:** Keith Morris-Schaffer, Alyssa Merrill, Katrina Jew, Candace Wong, Katherine Conrad, Katherine Harvey, Elena Marvin, Marissa Sobolewski, Günter Oberdörster, Alison Elder, Deborah A. Cory-Slechta

**Affiliations:** Department of Environmental Medicine, Box EHSC, University of Rochester Medical Center, Rochester, NY 14642 USA

## Abstract

**Background:**

Recent epidemiological studies indicate early-life exposure to air pollution is associated with adverse neurodevelopmental outcomes. Previous studies investigating neonatal exposure to ambient fine and ultrafine particles have shown sex specific inflammation-linked pathological changes and protracted learning deficits. A potential contributor to the adverse phenotypes from developmental exposure to particulate matter observed in previous studies may be elemental carbon, a well-known contributor to pollution particulate. The present study is an evaluation of pathological and protracted behavioral alterations in adulthood following subacute neonatal exposure to ultrafine elemental carbon. C57BL/6J mice were exposed to ultrafine elemental carbon at 50 μg/m^3^ from postnatal days 4–7 and 10–13 for 4 h/day. Behavioral outcomes measured were locomotor activity, novel object recognition (short-term memory), elevated plus maze (anxiety-like behavior), fixed interval (FI) schedule of food reward (learning, timing) and differential reinforcement of low rate (DRL) schedule of food reward (impulsivity, inability to inhibit responding). Neuropathology was assessed by measures of inflammation (glial fibrillary-acidic protein), myelin basic protein expression in the corpus callosum, and lateral ventricle area.

**Results:**

Twenty-four hours following the final exposure day, no significant differences in anogenital distance, body weight or central nervous system pathological markers were observed in offspring of either sex. Nor were significant changes observed in novel object recognition, elevated plus maze performance, FI, or DRL schedule-controlled behavior in either females or males.

**Conclusion:**

The limited effect of neonatal exposure to ultrafine elemental carbon suggests this component of air pollution is not a substantial contributor to the behavioral alterations and neuropathology previously observed in response to ambient pollution particulate exposures. Rather, other more reactive constituent species, organic and/or inorganic, gas-phase components, or combinations of constituents may be involved. Defining these neurotoxic components is critical to the formulation of better animal models, more focused mechanistic assessments, and potential regulatory policies for air pollution.

**Electronic supplementary material:**

The online version of this article (10.1186/s12989-019-0293-5) contains supplementary material, which is available to authorized users.

## Introduction

The theory that early-life exposure to air pollution, a complex mixture of particles, gases, and inorganic and organic contaminants, constitutes a risk factor for adverse neurodevelopmental outcomes is increasingly supported by human and animal studies. Epidemiological studies have linked air pollution with cognitive impairments [1, 2], reduced mental development index and IQ scores [3], attention-related disorders [4–6], anxiety/depression [7, 8], nonverbal reasoning deficits [9], autism spectrum disorder (ASD) [10, 11] and delayed psychomotor development [12]. In animal models, developmental exposure to ambient particulate matter produced depression-like phenotypes [13], reduction in exploratory behavior [14] and novel object recognition deficits [15], impulsivity-like phenotypes [16], and cognitive dysfunction [17]. The specific component(s) of air pollution underlying this neurotoxicity are not known.

Typically, particulate matter (PM) is classified by aerodynamic diameter, ranging from coarse (between 2.5 and 10 μm; PM_10_) to fine (< 2.5 μm; PM_2.5_) to ultrafine (< 100 nm or 0.1 μm; UFP). Ambient UFPs arise primarily from combustion processes, including the combustion of fossil fuels, of which motor vehicle traffic is the primary source [18]. Though not a significant contributor to air pollution in terms of mass relative to larger particles, UFPs achieve orders of magnitude higher particle numbers and surface areas that include toxic air pollutants (oxidant gases, organic compounds, and transition metals). The small size of UFPs produces more serious health consequences [19, 20], as they are deposited efficiently throughout the respiratory tract and absorbed into the bloodstream following inhalation exposure, then distributed to other organs, including the heart and brain [21]. Deposition of inhaled UFPs in the nasal cavity can lead to particle translocation to the brain, bypassing the blood brain barrier [22]. There is limited work on the developmental effects of pollutant-derived ultrafine particles on adverse neurodevelopmental outcomes. Neonatal exposure to concentrated ambient UFPs has been shown to induce impulsivity-like phenotypes, persistent microglial activation, disruption in excitatory/inhibitory balance, and neuropathological changes that are male-biased [16, 23–25]. This work specifically suggested some physiological plausibility linking early-life exposure to UFPs to poor health outcomes including ASD, which also have a strong male prevalence. However, these studies do not provide specificity on the constituents or sources of the ambient UFPs contributing to these adverse outcomes, which is necessary for enacting nuanced regulatory policies.

Current regulation in the U.S. targets the levels of PM_2.5_ and PM_10_, presuming that UFP levels will consequently also drop. Even with increasing regulation, the ongoing urbanization trends and expanding road traffic in many areas of the world are predicted to further increase population exposures to UFPs [26]. While the exact composition of air pollution varies in relation to geography, climate, season, traffic density, and other local factors, a primary constituent of ambient UFPs is carbonaceous material, reflecting the dominant combustion sources that generate these materials, including both elemental carbon (EC) and organic carbon (OC) [27–32]. EC/OC ratios vary depending upon conditions, but in cities such as Los Angeles, the percentage of OC ranged from 32 to 69% and EC from 1 to 34% [33]. Given its predominance as a major air pollution component, it is important to assess ultrafine elemental carbon’s potential in contributing to adverse neurodevelopmental outcomes.

Although elemental carbon is a commonly used surrogate in air pollution exposure studies [34–36], few animal studies have directly explored its potential CNS effects. Inhalation of ultrafine ^13^C particles in rodents resulted in translocation from the nasal epithelium to olfactory bulb, as well as to more distal regions, including the cerebellum [37] suggesting a potential route of neurotoxicity. While a single exposure of adult mice to pure ultrafine carbon particles generated via a spark-discharge method did not alter cytokine mRNA or protein levels in the CNS when measured immediately after exposure [38], intranasal instillation of 8 week old male mice with 14 nm Printex 90 carbon black particles induced pro-inflammatory cytokines IL-1β and TNFα, the chemokines monocyte chemoattractant protein-1/CCL2, and macrophage inflammatory protein-1α/CCL3 in olfactory bulb [39]. However, these particles are not exclusively elemental carbon and contain chemisorbed organic constituents, as well as other trace contaminants that may contribute to the phenotype [40, 41]. Furthermore an intranasal instillation delivers Printex 90 at a high dose and high rate, which is limited in predicting the effects of a subacute or chronic human inhalation exposure.

The present study investigates the developmental neurotoxic potential of ultrafine elemental carbon particles (UFCP) using a neonatal whole-body inhalation exposure model. A daily human-relevant exposure mass concentration was designed for mice based off cross-species particulate dosing calculations using Multiple-Path Particle Dosimetry software (MPPD). An exposure timescale, postnatal day (PND) 4–7 and 10–13 was based on multiple studies reporting adverse CNS pathology and protracted behavioral outcomes from subacute neonatal exposure to ambient ultrafine particles [23–25, 42–45]. A comprehensive battery of well-validated and translational behavioral assays was utilized to evaluate the developmental effects of UFCP on activity levels, recognition memory, fear-mediated behavior, social preference, temporal control, and impulsive action. Furthermore, biomarkers of toxicity were explored in the central nervous system and pulmonary system to evaluate UFCP’s effects on potential underlying physiological pathways.

## Results

### Exposure

**Fig. 1:**
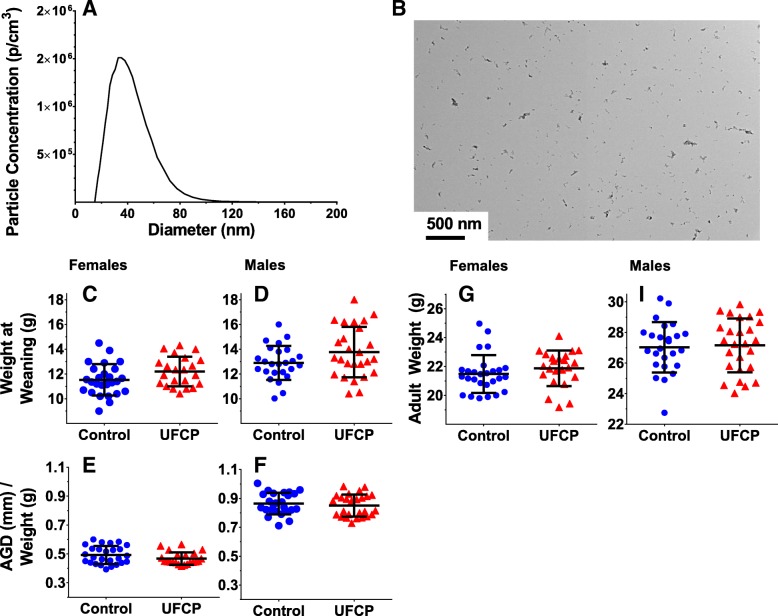
Exposure characterization, body weights, and anogenital distance. An example size distribution of the ultrafine carbon particles generated with the Palas spark generator (**a**). TEM images of ultrafine carbon particles collected via electrostatic precipitation on a carbon-coated copper grid (**b**). Offspring body weights (litter weight/number of pups) and AGD (normalized to body weight) in females (**c**, **d**) and males (**e**, **f**) at weaning (PND 26–27, *n* = 22–26 mice/sex/treatment group). Offspring body weight at adulthood (~PND 60) for females (**g**) and males (**i**). Data are presented as the average per litter data points with the group mean ± SD for each treatment group

A sample size distribution of UFCP aerosol collected during an exposure session is shown in Fig. 1a. The size distribution of the aerosol (count median diameter; CMD) across all exposure days was 36 ± SD 2.1 nm, while the average mass concentration was 45.9 ± SD 7 μg/m^3^. The average daily particle count was 2.69 × 10^6^ ± SD 1.3 × 10^5^ p/cm^3^.

### Body weights, and anogenital distance

Body weights at PND 26–27 (weaning) and PND 60 (adults) are reported for females (Fig. 1c, g) and for males in (Fig. 1d, i). Anogenital distance (AGD), normalized to weight, is reported for females (Fig. 1e) and males (Fig. 1f). Females exposed to UFCP had an increase in weight at weaning and adulthood (β = 0.273, SE = 0.160, *p* = 0.095, *n* = 25–26), although it failed to reach statistical significance and there was no interaction between PND and treatment. Furthermore, females exposed to UFCP had a decrease in AGD normalized to body weight at PND 26–27 (β = − 0.014, SE = 0.008, *p* = 0.080, *n* = 25–26), though it failed to reach statistical significance. No significant treatment-related differences or interactions were found for body weights in males or in AGD at PND 26–27.

### CNS pathology

**Fig. 2:**
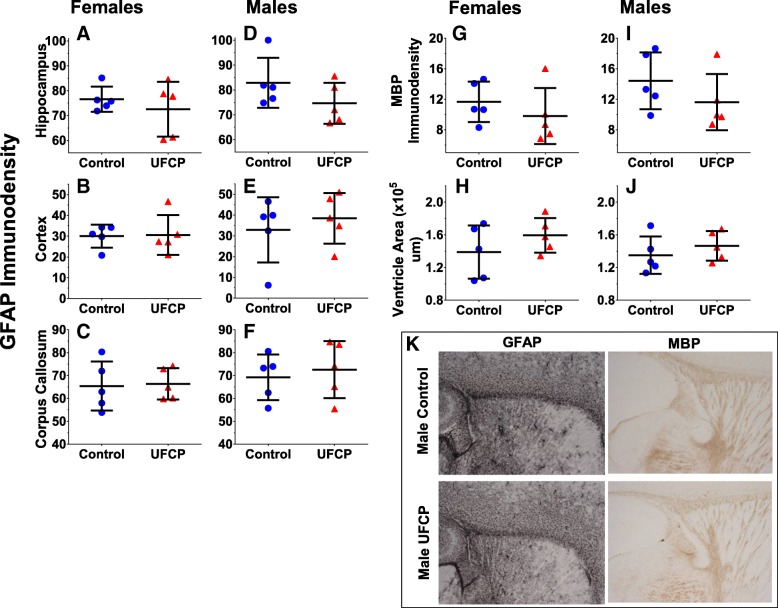
Regional GFAP immunoreactivity, MBP immunoreactivity, and ventricle area 24 h after exposure. Relative staining intensity of GFAP in the corpus callosum, cortex, and hippocampus respectively in females (**a**, **b**, **c**) and males (**d**, **e**, **f**). Relative staining intensity of MBP in the corpus callosum in females (**g**) and males (**i**). Average lateral ventricle area in females (**h**) and males (**j**). *n* = 5 mice/sex/treatment group. Example images of the GFAP and MBP staining are shown (**k**). Data are presented as individual data points with the group mean ± SD for each treatment group

The levels of glial fibrillary-acidic protein (GFAP) immunoreactivity in the corpus callosum, frontal cortex, and hippocampus are shown for females (Fig. 2a-c) and for males (Fig. 2d-f). No significant treatment-related differences were observed for GFAP immunostaining within the hippocampus, cortex, and corpus callosum for females or males. Levels of MBP (myelin basic protein) immunoreactivity with the corpus callosum and lateral ventricle area are reported for females (Fig. 2g, i) and males (Fig. 2h, j). As with GFAP, no significant treatment-related differences were observed for MBP immunostaining within the corpus callosum and no significant treatment-related differences in size of the lateral ventricles were found.

### Lung biomarkers

**Fig. 3:**
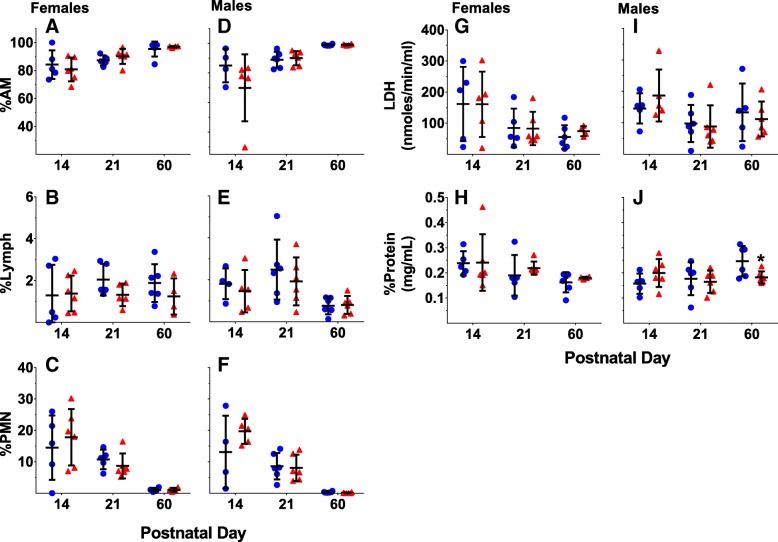
Pulmonary biomarkers of toxicity**.** Percentage of alveolar macrophages (AM), lymphocytes, and neutrophils (PMN) for females (**a**, **b**, c) and males (**d**, **e**, **f**). Lactate dehydrogenase (LDH) and protein content in females (**g**, **h**) and males (**i**, **j**). *n* = 4–6 mice/sex/treatment group. Data are presented as individual data points with the group mean ± SD for each treatment group. *p < 0.05 by student’s t-test

No significant treatment-related differences were found for percentage of alveolar macrophages (AM), lymphocytes (Lymph), and polymorphonuclear neutrophils (PMN) at any time point in either females (Fig. 3a-c) or males (Fig. 3d-f). No significant treatment-related differences in lactate dehydrogenase (LDH) content were observed at any time point in females (Fig. 3g) or males (Fig. 3i). For females there was a significant effect of time for %AM (β = 0.379, SE = 0.094, *p* < 0.001, *n* = 5), %PMN (β = − 0.294, SE = 0.048, *p* < 0.001, *n* = 5), LDH (β = − 1.529, SE = 0.709, *p* = 0.04, *n* = 5), and protein (β = − 0.001, SE = 0.0005, *p* = 0.036, *n* = 5). For males there was a significant effect of time for %AM (β = 0.379, SE = 0.093, *p* < 0.001, *n* = 4–6), %PMN (β = − 0.293, SE = 0.048, *p* < 0.001), %Lymph (β = − 0.026, SE = 0.008, *p* = 0.004), and protein (β = − 0.001, SE = 0.0004, *p* = 0.043). In females, no significant treatment related differences in protein content were found, although in males there was a significant interaction of treatment by time point (β = − 0.001, SE = 0.0004, *p* = 0.024). A post hoc student’s t-test showed significantly decreased protein content in UFCP-exposed males at PND 60.

### Locomotor activity

**Fig. 4:**
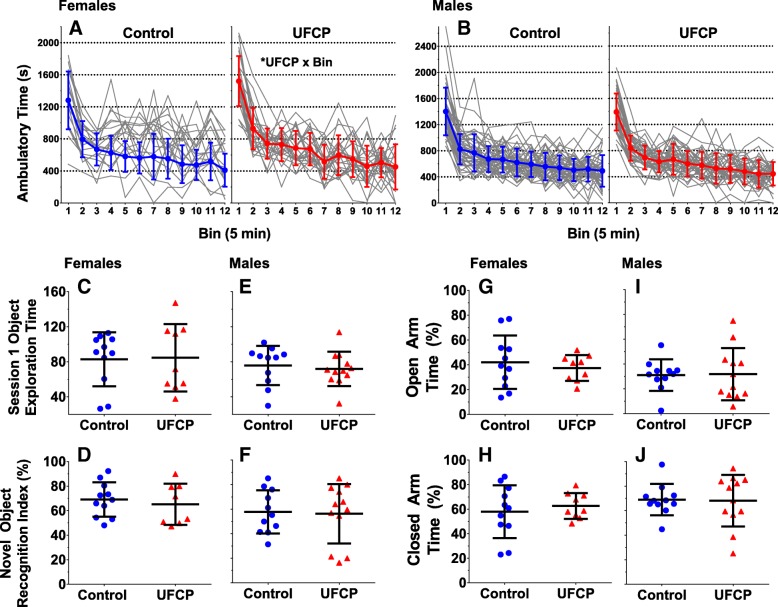
Locomotor activity and novel object recognition. Ambulatory time in 5 min epochs across one 60-min session for females (**a**) (*n* = 18–24) and males (**b**) (*n* = 37–46). Time spent with familiar objects in Session 1 and novel object recognition index in Session 2 for females (**c**, **d**) (*n* = 8–11) and males (**e**, **f**) (*n* = 11–12). Time spent in open arm and closed arm (percentage of total time in arms) on the elevated plus maze for females (**g**, **h**) (*n* = 8–11) and males (**i, j**) (*n* = 11–12). For the locomotor assay data are presented as individual learning curves with the group mean ± SD for each treatment group. For the novel object and elevated plus maze data are presented as individual data points with the group mean ± SD for each treatment group

Time spent in ambulation by mice across the 60 min session is reported for females (Fig. 4a) and for males (Fig. 4b). UFCP-exposed females had a significantly steeper slope (β = − 8.162, SE = 3.510, *p* = 0.025, *n* = 18–24) than controls and no significant treatment-related difference for average ambulatory time. For males (*n* = 37–46) no significant differences between UFCP-exposed mice and control mice were found in either habituation or average ambulatory time.

### Novel object recognition (NOR)

**Fig. 5:**
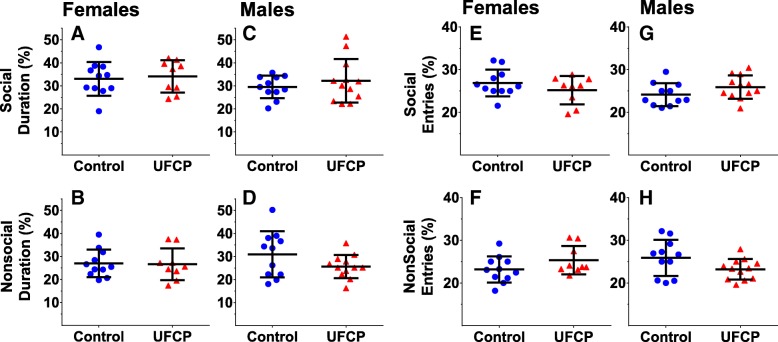
Social Conditioned Place Preference. Time spent in social chamber and nonsocial chamber as a percentage of the total duration for females (**a**, **b**) (*n* = 9–11) and males (**c**, **d**) (*n* = 11–12). Total social entries and nonsocial entries as a percentage of the total number of entries for females (**e**, **f**) and males (**g**, **h**). Data are presented as individual data points along with group mean ± SD for each treatment group

Time spent with original objects during the first session and the recognition index from the second session is reported for females (Fig. 4c, d) and for males (Fig. 5e, f). In neither case were significant treatment-related differences found between the UFCP-exposed mice compared to control mice in exploration time in the first session or for recognition index in either females (*n* = 9–11) or males (*n* = 11–12) in session 2.

### Elevated plus maze performance (EPM)

Time spent in the closed and opens arms of the elevated plus maze are reported for females (Fig. 4g, h) and for males (Fig. 4i, j). There were no significant treatment-related differences in females (*n* = 9–11) or males (*n* = 11–12) in the time spent in the open or closed arms.

### Social conditioned place preference (SCPP)

Time spent in social chamber and nonsocial chamber as a percentage of the total duration for females are reported (Fig. 5a, b) and for males (Fig. 5c, d). Total social entries and nonsocial entries as a percentage of the total number of entries females is reported (Fig. 5e**,** f) and for males (Fig. 5g, h). No significant treatment-related differences in females (*n* = 9–11) or males (*n* = 11–12) were found on any of the endpoints.

### Fixed-interval (FI) 60s schedule performance

**Fig. 6:**
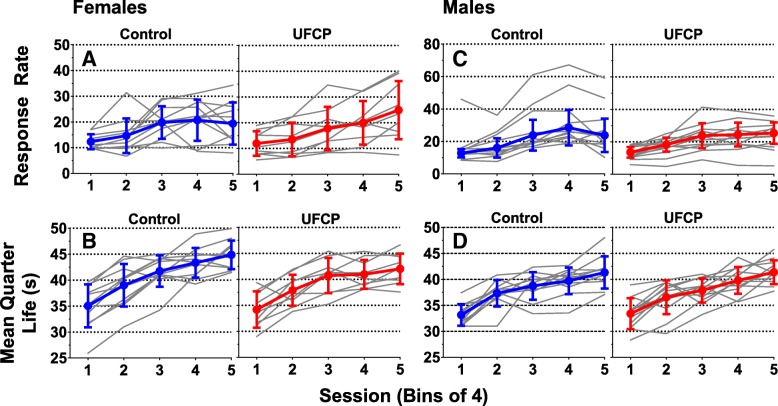
Fixed interval schedule of reward. Group mean response rates and mean quarter life values for females (**a**, **b**) (*n* = 9–10) and males (**c**, **d**) (*n* = 11–12). Data are presented as individual learning curves along with the group mean ± SD responses per interval or mean quarter life for bins of 4 sessions

Response rate and mean quarter life (MQL) values from the FI schedule of reward are shown for females in Fig. 6a, b and for males in Fig. 6c, d. There were no significant treatment-related differences in average response rate or in rate of learning (slope across sessions) for either sex. Females exposed to UFCP did show a decrease in average mean quarter life across sessions, but it failed to reach statistical significance (β = − 1.069, SE = 0.569, *p* = 0.078, *n* = 9–10). There was no significant treatment-related difference for mean quarter life slope in females. Additionally, there were no significant differences in either average response rate or learning slope in males, *n* = 11–12.

### Differential reinforcement of low rate (DRL) schedule performance

**Fig. 7:**
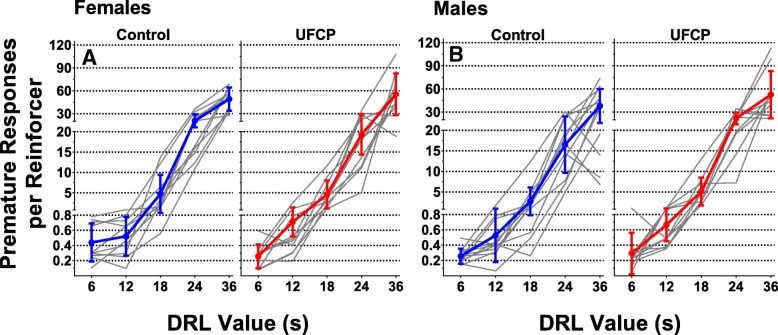
Differential reinforcement of low rates schedule performance. Premature responses per reinforcer for females (**a**) (*n* = 10) and males (**b**) (*n* = 10–12) across increasing time values on the DRL schedule. Data are presented as individual learning curves with the group mean ± SD premature responses per DRL value

Average premature responses per reinforcer are reported for females in (Fig. 7a) and for males (Fig. 7b). There were no significant treatment-related differences in numbers of premature responses at any of the required DRL time values for females (*n* = 10) or males (*n* = 10–12).

## Discussion

Positive correlations are observed between elevated PM levels and adverse neurodevelopmental outcomes including ASD [10, 11], attention-related disorders [4–6], and general cognitive decline [1–3, 9, 12]. Complementary developmental rodent models of exposure to ambient PM have further supported the plausibility that PM can lead to protracted behavioral and pathological alterations [16, 23–25, 43]. However, the exact constituents within PM that are contributing to the observed developmental phenotypes are still unclear. One common constituent with PM is elemental carbon, an inherent byproduct of engine combustion, present in urban areas in the United States and Europe [27–32]. Inhalation of UFCP using a spark discharge setup has been shown to lead to translocation of UFCP to the olfactory bulb, cerebrum, and cerebellum with UFCP retention within the cerebrum and olfactory bulb persisting for up to a week post-exposure [37]. The significant extent to which UFCP translocated to the CNS suggested a potential pathway for induction of developmental neurotoxic effects. The present study assessed the effects of repeated neonatal inhalation exposure (PND 4–7 & PND 10–13) to a high, but relevant concentration of UFCP on CNS pathology and different behavioral phenotypes. The exposures did not yield any evidence of pathological effects in response to UFCP as measured 24 h following exposure. The evidence for behavioral toxicity of UFCP was limited with no significant behavioral alterations beyond a significant treatment-related difference with activity habituation in females. Considered collectively, the findings demonstrate subacute neonatal exposures to UFCP do not appear to induce neuropathological, protracted pulmonary, or behavioral changes.

Previous studies have shown neonatal exposure to ambient ultrafine particles in an equivalent time frame can lead to male-biased alterations in neuroinflammatory biomarkers, white matter damage, and ventriculomegaly within the CNS [15, 23, 25].The observed male-biased pathologies had relevance as they are associated with symptoms often seen in children in male-prevalent disorders such as ASD [46–48] and ADHD [49, 50], the same disorders linked to adverse neurodevelopmental outcomes from early-life exposure to pollution particulate [6, 10, 11]. Despite the evidence that UFCP can effectively infiltrate and be retained in the CNS [37], the present study, using similar biomarkers of analysis as previous ambient studies, did not demonstrate any of those pathologies in either females or males suggesting UFCP is not a primary underlying contributor to the effects of ambient ultrafine particles. One limitation of the present study is the CNS pathology was explored only 24 h after the last day of exposure and there is the capacity for “silent” neurotoxicity in which an adverse series of underlying key molecular events is initiated during exposure, but the final pathological changes only manifest in the adolescent or adult following the completion of development. However, ambient developmental PM exposure models have shown adverse neuroinflammatory-like pathologies 24 h or less following the last day exposure [23, 25] and UFCP does not recapitulate those overt effects. Additionally the lack of functional behavioral outcomes across a comprehensive-battery of paradigms does not suggest any detrimental pathology manifested during adulthood.

Beyond CNS pathologies, this study also explored pulmonary outcomes as a secondary endpoint. A hypothesized mechanism by which UFCP can influence the CNS indirectly is via continuously stimulated effects by retained particles in alveolar macrophages and epithelial cells which can produce pro-inflammatory cytokines, chemokines, and reactive oxygen species that can enter systemic circulation and trigger distal signaling events. The null pulmonary toxicity results at PND 14 and PND 21 in males do not suggest either an immediate or delayed inflammatory response to UFCP. It is worth addressing that the high PMN counts at PND 14 and PND 21 in both the controls and the UFCP mice relative to the adult time points is likely due to the integrity of the air-blood barrier at the early-stage development and not suggestive of inflammation. The null data does complement a previous study in which male Fischer 344 rats were exposed acutely to 150 μg/m^3^ UFCP, resulting in no pulmonary toxicity when examined 24 h after exposure [51]. Interestingly, there was a significant UFCP-mediated decrease in lung protein content in males only at PND 60, though the underlying mechanism is unclear and it may be spurious given no other biomarkers at the time point were influenced. Further breakdown of the protein content and its constituents may help elucidate the origin of the effect. However, neonatal UFCP exposure does not appear to elicit overt pulmonary toxicity.

To explore the neurobehavioral functional effects of developmental UFCP exposure, one squad of mice engaged in a series of highly standardized behavioral assays, including locomotor for activity levels, novel object for recognition memory, and elevated plus maze for an anxiety-like phenotype. Previous studies have shown neonatal exposure to ambient ultrafine PM can alter habituation and novel object discrimination [17, 25]. Additionally, several gestational fine PM exposure studies have shown increased anxiety-like phenotype on the elevated plus maze [52–54]. Interestingly, females showed a significantly steeper habituation slope on the locomotor assay that seemed to be derived from increased activity levels during the early stages of the assay. The underlying pathway(s) to that behavioral outcome are unclear and the behavior did not generalize to increased activity levels on the more active learning paradigms, the FI and DRL schedule. With regards to the novel object and elevated plus maze, developmental exposure to UFCP did not lead to any substantial treatment-related differences. In terms of behavioral quality assurance, the male and female controls, despite experiencing substantial maternal separation during development, did display the expected behavioral phenotypes on novel object and elevated plus maze giving more credibility to the null findings. Females and male controls had an average of 69 and 58% recognition index respectively on the novel object assay indicating a preference for exploring the new object. Furthermore, female and male controls spent substantially more time in the closed arms at an average of 47 and 53% respectively than the open arms, 34 and 24% respectively, indicating an aversion to the exposed arms.

One of the primary disorders linked to early-life exposure to traffic-related PM is ASD [10, 11, 55], a phenotype characterized in part by deficiencies in social outcomes. The epidemiological research has been complemented by developmental rodent exposures to ambient fine and ultrafine particles which, induce protracted alterations in social behavior [15, 42, 56]. To explore the potential contribution of developmental exposure of UFCP on social behavior a conditioned place preference paradigm was utilized which, is a well-validated translation assay used in human children [57]. Interestingly, no significant UFCP treatment-related differences were found in terms of social or nonsocial chamber preference. The potential reasons for the lack of effects are disruptions in social preference and social interactions from PM are often linked to early-life exposure to endocrine active chemicals which is not an expected property of UFCP. It is worth noting the social conditioned place preference assay’s interpretation is restricted to social motivation and more in-depth social experiments would need to be done to assess potential alterations in social interactions.

Beyond social dysfunction, restrictive and repetitive behavioral patterns are also an expected phenotype of ASD. In order to evaluate the effects of UFCP on those cognitive domains, two schedules of reinforcement, fixed interval and differential reinforcement of low rates were run. Self-control and delayed-gratification in young children has been linked to effective temporal recognition and responses on a FI schedule giving the assay translational validity [58, 59]. Moreover, neonatal exposure to ambient ultrafine particle at varying mass concentrations has shown to consistently influence FI behavior [25, 44]. However, no treatment-related developmental effects of UFCP on the FI schedule were found in the present study. Complementing the FI schedule, the DRL paradigm not only assesses temporal recognition but also impulsive action, the ability to inhibit consequential responses which the DRL punishes through resetting of reinforcement timer. As expected in the controls, when the time to inhibit responding increased, the task became more difficult, the amount of premature responses per reinforcer increased but there were no significant treatment-related changes. The non-significant result is somewhat interesting as children’s exposure to pollution-attributed elemental carbon has been associated with behavioral outcomes linked to impulsive behaviors [5, 60]. However, the data suggests elemental carbon in itself is not developmentally neurotoxic and it is more likely other pollution byproducts, such as polycyclic aromatic hydrocarbons (PAHs) or metals associated with elemental carbon concentrations are contributing to adverse developmental outcomes.

One major limitation of using spark discharge-generated UFCP is that they are only one component – physically and chemically – of the carbonaceous aerosols released during incomplete combustion from gasoline and diesel fuel-burning sources [41, 61]. However, as the goal of this study was to assess the contribution of EC alone to developmental neurotoxicity, spark discharge methods provide a reliable and verifiable means of ultrafine exposure [61]. Another limitation of the study is the calculated equivalent dose via MPPD, alveolar surface deposition, may not be the most relevant dose with regards to the CNS, given the alternative neuronal pathway by which ultrafine particles deposited on the nasal epithelium can access the CNS. It is also worth noting the MPPD reference elemental carbon exposure concentration for humans, 5 μg/m^3^, is based on the mass fraction of total elemental carbon content in urban environments which, is not exclusively ultrafine particles, thus there may be an overestimation of the fully realized human alveolar surface deposition dose. With regards to mouse exposure conditions, all pups underwent repeated maternal separation and handling, stress-inducing factors [62, 63] that can alter physiological responses to subsequent stressors [64, 65]. However, given that repeated maternal separations persistent effects on offspring behavior is mixed, with null [66–68] and positive [69–71] findings, it is unlikely all null findings within this study are solely due to repeated maternal separation overriding particle effects. Lastly, although this study primarily shows null findings, there still remains the potential for gestational exposure to UFCP as well as chronic exposure throughout the postnatal, juvenile, and adult window to exert adverse neurodevelopment outcomes.

A potential reasons UFCP did not exert any overt toxicological effects is UFCP does not contain the more bioactive redox-cycling metals, soluble organic species, and semi-volatiles that would otherwise be present in an ambient exposure. In fact, several epidemiological studies more directly associate ambient metal content [72–74], PAHs [75, 76], and volatile organic species [77, 78] with a variety of adverse neurodevelopmental outcomes. Furthermore the studies showing ultrafine carbonaceous particles induce an inflammatory or oxidative stress reaction within the CNS use Printex 90 particles, a more soot-like particle with known chemisorbed constituents, which the present study suggests may be the underlying toxic contributors rather than the elemental carbon fraction alone [39, 40, 79]. Despite the notion that UFCP has some biochemical capacity through its highly energetic and reactive surface area, the data in the study suggests neonatal CNS contains sufficient protective mechanisms to prevent an over reactive neuroinflammatory response to UFCP exposure alone. One of the primary public health concerns of ultrafine particles is their capacity to infiltrate the developing CNS but, the data presented in this study suggests regulators need to be as concerned with the underlying constituents in order to properly evaluate risk.

## Conclusion

In summary, subacute neonatal exposure to spark-discharged generated UFCP did not induce any early adverse pathology or protracted behavioral dysfunction in adulthood. The only significant observed behavioral effect of UFCP was a habituation alteration in females, though it is uncertain about the underlying behavioral and physiological mechanisms of this effect. Further reductionist studies of particulate matter with regards to air pollution may benefit from exploring potentially more potent contributors, such as PAHs, semi-volatile and volatile species, and reactive metals.

## Methods

### Breeding and exposure

Eight-week-old male and female C57BL/6J mice were purchased from Jackson Laboratories (Bar Harbor, ME) and allowed to acclimate in the housing room for one week prior to pairing. All mice in this study were housed under a 12 h reversed light/dark cycle and temperature maintained at ~ 22 °C. Three days prior to pairing, dirty male bedding was added to the female cages to synchronize estrous cycles (Whitten effect) and increase the likelihood of impregnation. Monogamous pairs of mice were bred for three days; males were then removed and dams remained singly-housed with litters until weaning. Litters were randomly allocated into the control and exposed groups. Neonatal mice were separated from the dams for exposures, which occurred from postnatal days (PND) 4–7 and PND 10–13 for 4 h/day between 1000 and 1400 h. During these exposures, the mice pups were housed in small mesh chambers with 2–4 pups per chamber. The mice pups were returned to the original dam upon completion of each exposure session. To address litter-specific effects, only one male and female mouse was used per litter per treatment group per endpoint and the primary unit of sample variability reported in the analyses, *n*, refers to the litter. All experimental activities were approved by the University of Rochester Institutional Animal Care and Use Committee.

Using a Palas spark generator (Palas Model GFG-1000, Karlsruhe, Germany), UFCP were produced in an argon-filled chamber using electric arc discharge between two opposing ultrapure graphite rods (ESPI Metals, Ashland, OR) and electrostatic charge was brought to Boltzmanm equilibrium by a ^210^Po source. Exposure conditions are based on modeling calculations (MPPD v.3.04; Additional file 1) to produce equivalent particle deposition per alveolar surface area in the mice with a 4 h exposure to that of a human infant exposed over 24 h. The targeted human concentration was 5 μg/m^3^, a relatively high human exposure, with elemental carbon ambient content in the United States and Europe ranging from 0.25–3.5 μg/m^3^ [27–32]. After exiting the chamber, the carbon UFCP were mixed with filtered, medical-grade air and entrained at a rate of 25–30 L/min into a 60 L stainless steel-reinforced Lexan exposure chamber housing the neonatal mice. Both UFCP and control mouse chambers were maintained at 22–25 °C and 35–40% relative humidity. UFCP aerosol had a particle number concentration of 3 × 10^6^/cm^3^ with count median diameter (CMD) of 35–40 nm and geometric standard deviation (GSD) of 1.4–1.5 (Model 3022 condensation particle counter with model 3071 differential mobility analyzer; TSI, Inc., St. Paul, MN). The mass concentration was monitored gravimetrically. The exposure goal was an average value of 50 μg/m^3^ UFCP. Control mice were exposed to high efficiency particulate (HEPA) filtered medical-grade air. At weaning, PND26–27, the weight and anogenital distance (AGD) was evaluated in male and female offspring.

For imaging, UFCP particles were collected via electrostatic precipitation on carbon coated copper grids (CF-200CU, Electron Microscopy Sciences) which, were imaged using a Hitachi 7650 Analytical TEM with an Erlangshen 11 megapixel digital camera and Gatan software.

### Time scale and endpoint overview

Refer to Table 1 for an overview of the exposure and endpoint time points. Three separate squads of mice were utilized for the behavioral assays to mitigate the extraneous consequences of previous behavioral experience influencing outcomes on a subsequent assay.

### CNS pathology

On PND 14, mice were euthanized by rapid decapitation. Brains were extracted and placed in 4% paraformaldehyde for 24 h, then into 30% sucrose until sunk. The brains were sectioned on the sagittal plane on a freezing microtome (Microm HM 440 E; GMI Inc., Ramsey, MN) at 40-μm thickness in cryoprotectant (30% sucrose, 30% ethylene glycol in 0.1 M phosphate buffer) and stored at − 4 °F (− 20 °C) until immunostaining. Every sixth section was stained for glial fibrillary acidic protein (GFAP) and myelin basic protein (MBP). GFAP is a filament component of astrocytes and is typically upregulated during neuroinflammation and neurotrauma [80, 81]. MBP is an abundant protein found in myelin sheaths which, plays a critical role in establishing the structural and functional properties of myelin [82]. Briefly, the brain sections were washed to remove cryoprotectant and placed into primary antibody solutions for GFAP (AB5804, 1:3000 dilution; Millipore, Billerica, MA) or MBP (MAB386, 1:1000; Millipore) for 24 h. For GFAP, the tissue was then placed into a biotinylated anti-rabbit IgG antibody solution (BA-1000, 1:200 dilution; Vector Labs, Burlingame, CA) and for MBP, a biotinylated anti-rat, mouse-absorbed, IgG antibody (BA-9401, 1:200 dilution; Vector Labs) for 1 h and the stain was visualized using DAB diaminobenzidine hydrochloride (D0426, SIGMA FAST DAB with metal enhancer, Sigma Aldrich, St. Louis, MO). Immunolabeled tissue was mounted onto Superfrost Plus micro slides (48311–703, VWR, Radnor, PA) and cover-slipped using Cytoseal 60 (23–244,257, Fisher Scientific, Pittsburg, PA).

Slide-mounted tissue sections were visualized on an Olympus BX41 microscope (Olympus America, Inc., Central Valley, PA) mounted with an MBF CX9000 camera (MBF, Villiston, VT) for GFAP and MBP image capture. To quantify MBP and GFAP immunostaining intensity, images were captured at 40x at the same exposure and brightness settings, and analyzed by optical densitometry using NIH ImageJ software. Optical density was deduced from mean grayscale analyses of the region of interest (ROI) standardized to a negatively stained region within the same section to normalize background grayscale. Three measurements taken on 3 separate sections per brain were performed per region of interest (ROI). These measurements were incorporated into a mixed model to explore the average change in staining across the sections (described in Statistical Analysis). The ROIs for MBP and GFAP immunostaining in the corpus callosum and frontal cortex were bregma 2.64–3.00 mm and GFAP immunostaining in the hippocampus at bregma 2.88–3.44 mm. The results are expressed as the difference between the background greyscale value and the positively-stained region, with higher values demonstrating increased immunoreactivity. Lateral ventricle area tracings were performed using Neurolucida software (MBF, Williston, VT) with 3 consecutive sections used per subject. A murine anatomical brain atlas was utilized in conjunction with identification of anatomical landmarks to ensure sections of homologous bregma were analyzed [83].

### Pulmonary biomarkers

On PND 14, 21, and 60 mice were euthanized with an overdose of Euthasol followed by exsanguination. The lung/heart block was excised and excess tissue removed prior to the lungs being lavaged with sterile, 0.9% saline (0.3 mL), keeping the first two lavage supernatants separate from the remaining following centrifugation (10 min, 350 x g, 4 °C). Bronchoalveolar lavage (BAL), cell viability (trypan blue exclusion), number (hemacytometer), and the percentages of polymorphonuclear neutrophils (PMN), alveolar macrophages (AM), and lymphocytes (Lymph) (Hema 3®; Fisher Scientific, Kalamazoo, MI) were determined. Total protein concentration was measured as an indicator of cytotoxicity and epithelial barrier permeability with the bicinchoninic acid (BCA) assay using reagents purchased from Thermo Scientific (Rockford, IL). Lactate dehydrogenase (LDH) as an indicator of cell membrane integrity was determined using reagents from Sigma.

### Locomotor activity

Spontaneous locomotor activity was measured in photobeam chambers equipped with a transparent acrylic arena with a 48-channel infrared source, detector, and controller (Med Associates, St. Albans, VT). Locomotor activity was quantified in one 60-min session, with the primary endpoint, ambulatory time, collected in 5 min epochs. Ambulatory time was defined as the cumulative time in which there were successive breaks of 2 × 2 photobeam virtual boxes within the chamber. A period of ambulation is stopped when the mouse remained within a 2 × 2 virtual box for ≥300 ms. Habituation and average ambulatory activity in each session were explored. For analysis, data from the two squads was combined given the mice were run on the same time points and had equivalent prior behavioral and exposure experience (Table 1). A squad of extra males was included for the locomotor assay at the same time point, thus the final sample sizes for males is *n* = 37–46, while the female sample size is smaller *n* = 18–24.

### Novel object recognition

Novel object recognition testing consisted of two phases and was conducted in an open Plexiglas arena (dimensions: 30.5 cm × 30.5 cm × 30.5 cm). In the first session, mice were placed for 10 min in the test environment, which contained the two familiar objects. The two familiar objects were smooth, marble white door knobs. During the session, side preference, exploration time, and patterns of exploration among treatment groups were assessed. In the second session, occurring 24 h after session one, mice were returned to the arena for 5 min during which a novel object (black marble door knob) now replaced one of the previous two objects. Placement of the familiar versus novel object was counterbalanced across treatments and subjects to preclude bias. All sessions were videotaped and scored using Observer XT 13.0 (Noldus) by a reviewer blinded to treatment group. Exploration was defined as a mouse oriented toward the object with head first entry into a pre-marked 2 cm circle surrounding the object. A recognition index was calculated based on the following equation.

$$ Recognition\kern0.17em Index=\frac{Time\kern0.17em with\kern0.17em Novel\kern0.17em Object}{Time\kern0.17em with\kern0.17em Novel\kern0.17em Object+ Time\kern0.17em with\kern0.17em Familiar\kern0.17em Object}\times 100 $$

### Elevated plus maze

Mice were placed on an elevated plus maze apparatus, which included open (with no sides) and closed (with sides) arms for a 5 min period during which time spent in the open versus closed arms was measured. Sessions were video-recorded and the videos scored by a blinded observer using Observer XT 13.0 (Noldus). The primary endpoints are the total time spent in the open arms and closed arms over the total session time.

### Social conditioned place preference

SCPP testing occurred in a three-chambered polycarbonate box (63 cm × 27 cm × 31 cm). One chamber was wall papered with vertical and a second chamber was wall papered with horizontal black and white stripes. In a manner counter-balanced across individuals, both for side and paper orientation, one chamber was designated as the social chamber, while the other was designated as the solitary chamber. Testing consisted of two phases, training and preference testing. Training occurred for 10 min a day for 10 days. During this training period, mice could choose between the side chamber that contained its cage mate (placed under a wire cup) or the side chamber that contained only an empty wire cup. For the preference test, both side chambers were empty, and mice were returned to the center arena, and their subsequent preference behavior videotaped and scored for the next 5 min. Data collected include total duration in each chamber and number of entries into each chamber. A Social Chamber Preference Index was calculated using the following equation:

$$ Social\ Chamber\ Preference=\frac{Social\ Duration\ or\ Counts}{\left( Center+ Nonsocial+ Social\right)}\times 100 $$

#### Food restriction and lever training for operant paradigms

To enhance and normalize motivation for a food-reinforcement, mice were placed on a food-restricted schedule for 3 days immediately prior to initiation of operant training to reach 85% of ad libitum weight and were maintained at 85% ad libitum body weight throughout the period of operant behavior assessment. Testing was conducted in operant chambers (Med Associates, St. Albans, VT) housed in sound-attenuating cabinets equipped with white noise and fans for ventilation. Three levers were located horizontally across the back wall of the chamber, with a pellet dispenser for reinforcer delivery on the front (opposite) wall. Mice were initially trained to press a lever for food reward using a variable time 60 s fixed ratio 1 schedule (VT60FR1), in which a reinforcer (20 mg food pellet) was delivered simultaneously with a light and sound cue on average every 60 s independently of behavior. A response on the designated correct lever during this period would also trigger the light and sound cue, and reinforcement delivery. Following ten correct lever press responses or a total of 20 min on the VT60 component, the schedule was changed to a fixed ratio 1 schedule which, required a lever press on the designated correct lever for each food delivery until fifty reinforcers had been delivered. As behavioral experience can heavily modify behavioral output, two separate cohorts of animals were trained and used independently for the fixed interval schedule and differential reinforcement of low rates schedule.

### Fixed interval schedule

After lever press training was completed in all mice, the schedule was shifted to a 60 s FI schedule (FI60) examined in 30 min sessions over a total of 20 consecutive sessions to assess learning. On the FI schedule, the first lever press response on the designated correct lever after completion of a 60 s interval produced food delivery and initiated the next 60 s interval until 30 min had elapsed. Responses during the interval itself had no programmed consequence. Measures of FI performance included response rate (total responses/total session time) and quarter life (the latency from the onset of an interval to the time at which the first one quarter of the responses in the interval occurred to assess temporal control). Initially, performance on the FI schedule is characterized by uniform responding throughout the interval. However, over sessions, as temporal control is established, pausing begins to follow reinforcement delivery and maximal responding shifts to later in the interval, with quarter life values thus initially increasing significantly over early sessions, followed by more gradual but, continual increases as behavior stabilizes. A mean quarter life (MQL) value was generated across the intervals in each session.

### Differential reinforcement of low rate schedule

Paradigm for mice described previously [17]. After lever press training was completed in all mice, it was followed by implementation of the DRL schedule of reinforcement. The DRL schedule reinforces responses separated by a designated inter-response time. Similar to the FI, the schedule required a specified length of time to elapse before a response could occur to produce food reward. Premature responses, i.e., those occurring prior to the end of this interval value reset the timer. The initial DRL value was set at 6 s and was subsequently increased to 12, 18, 24, 30, and 36 s across sessions. Behavioral test sessions were 30 min in length, with 3 sessions at 6 s, 4 sessions at 12 s, 5 sessions at 18 s, 4 sessions at 24 s, and 9 sessions at 36 s. Performance on the DRL schedule was evaluated using the number of premature responses per reinforcer/pellet, a measure of response efficiency.

### Statistical analysis

All pathological and behavioral analyses were stratified by sex. The novel object recognition paradigm, and elevated plus maze data were analyzed by student’s t-test. The pulmonary markers were analyzed with a two-way ANOVA with treatment and postnatal day as the two fixed factors. The CNS pathology, ambulatory activity, fixed-interval performance data were analyzed using a mixed model approach as previously described [44]. A random intercept and when necessary, a random slope component, were used to capture individual-level variability on performance and learning. For ambulatory activity and the fixed interval schedule, the intercept component was centered; subtracting the overall mean from every value of a variable, ensuring the mean has a value of 0, and used to explore the average response across the sessions, while a slope was used to define the linear function of learning across the sessions. UFCP exposure and session were designated as the fixed effects in the model, and the differences in average response rate and learning slope were compared between treatment groups. A random intercept-only model was used to examine the average number of premature responses per session for each DRL value for the DRL schedule. Each inter-response time analysis was conducted independently. For mixed model findings, the estimates (β), standard errors (SE), and *p*-values are reported. The a priori statistical significance criterion was α ≤ 0.05, though *p*-values ≤0.1 are also noted in the results. All analyses were conducted using JMP Pro 13.0 (SAS Institute Inc., Cary, NC).

## Additional file

Additional file 1: Supplementary Methods Multiple-Path Particle Dosimetry. (DOCX 20 kb)

Additional file 2: UFCP Experiment Dataset. (XLSX 631 kb)
