# Supplementary material for: Effects of neonatal inhalation exposure to ultrafine carbon particles on pathology and behavioral outcomes in C57BL/6J mice
Source: Part Fibre Toxicol. 2019 Feb 18;16:10. doi: 10.1186/s12989-019-0293-5 (PMC6379948; doi:10.1186/s12989-019-0293-5)
Supplement: Supplementary file 1 — Supplementary Methods Multiple-Path Particle Dosimetry. (DOCX 20 kb) [file 12989_2019_293_MOESM1_ESM.docx]

**Supplementary Methods**

*Use of Multiple Path Particle Dosimetry (MPPD) to calculate human equivalent concentration from a mouse exposure scenario*

Human modeling data from the MPPD program (version 3.04) using the 3-month old 5-lobe model, in a nasal breather under constant exposure were selected. The diameter of spark-discharge UFCP was 40 nm with a 1.5 GSD and the targeted aerosol concentration for humans was 5 μg/m^3^. For the 3-month old model, the alveolar surface area deposition across 24 hours was ~0.0638 ng/cm^2^. Neonate mouse modeling data were obtained from background literature and the default adult mouse lung data overwritten when possible to correct for the age-related physiological parameters. The particle properties were the same as the human and the targeted aerosol concentration for mice was 50 μg/m^3^. For the mouse neonate model across 4 hours, the alveolar surface area deposition was~0.0696 ng/cm^2^.

**Human 3 month-old, 5-lobe model (default parameters)**

a) Functional residual capacity = 17.97 mL

b) Upper respiratory tract volume = 2.45 mL

c) Breathing frequency = 39 breaths/min

d) Tidal volume = 30.44 mL

c) Inspiratory fraction = 0.5

**Neonate mouse parameters**

a) Functional residual capacity = 0.130 mL [74]

b) Upper respiratory tract volume = 0.01 (scaled down from default adult mouse, 20 g, URT = 0.0322)

c) Breathing frequency = 200 breaths /min [75]

d) Tidal volume = 60 μL [75]

c) Inspiratory fraction = 0.82 [76, 77]

1. Bozanich EM, Janosi TZ, Collins RA, Thamrin C, Turner DJ, Hantos Z, Sly PD: **Methacholine responsiveness in mice from 2 to 8 wk of age.** *J Appl Physiol (1985)* 2007, **103:**542-546.

2. Bissonnette JM, Knopp SJ: **Developmental changes in the hypoxic ventilatory response in C57BL/6 mice.** *Respiration Physiology* 2001, **128:**179-186.

3. van Schaik SM, Enhorning G, Vargas I, Welliver RC: **Respiratory syncytial virus affects pulmonary function in BALB/c mice.** *J Infect Dis* 1998, **177:**269-276.

4. Sozo F, Horvat JC, Essilfie A-T, O’Reilly M, Hansbro PM, Harding R: **Altered lung function at mid-adulthood in mice following neonatal exposure to hyperoxia.** *Respiratory Physiology & Neurobiology* 2015, **218:**21-27.
